# Algal Viruses: The (Atomic) Shape of Things to Come

**DOI:** 10.3390/v10090490

**Published:** 2018-09-12

**Authors:** Christopher T. Evans, Oliver Payton, Loren Picco, Michael J. Allen

**Affiliations:** 1 Plymouth Marine Laboratory, Plymouth PL1 3DH, UK; chev@pml.ac.uk; 2 Interface Analysis Centre, Wills Physics Laboratory, University of Bristol, Bristol BS8 1TL, UK; oliver.payton@bristol.ac.uk (O.P.); loren.picco@bristol.ac.uk (L.P.); 3 Department of Physics, Virginia Commonwealth University, Richmond, VA 23284, USA; 4 College of Life and Environmental Sciences, University of Exeter, Exeter EX4 4QD, UK

**Keywords:** algal virus, imaging, atomic force microscopy, electron microscopy, virus structure, infection dynamics

## Abstract

Visualization of algal viruses has been paramount to their study and understanding. The direct observation of the morphological dynamics of infection is a highly desired capability and the focus of instrument development across a variety of microscopy technologies. However, the high temporal (ms) and spatial resolution (nm) required, combined with the need to operate in physiologically relevant conditions presents a significant challenge. Here we present a short history of virus structure study and its relation to algal viruses and highlight current work, concentrating on electron microscopy and atomic force microscopy, towards the direct observation of individual algae–virus interactions. Finally, we make predictions towards future algal virus study direction with particular focus on the exciting opportunities offered by modern high-speed atomic force microscopy methods and instrumentation.

## 1. Introduction

Considering the sheer diversity and abundance of algal viruses [1,2], and their influence in global geochemical cycles, potential food and value product stocks, and even climate change [3,4]; the quantity of research into their physical and structural characteristics, and its implications for their infection strategies is surprisingly low. Gaining structural insight may seem of little importance to understanding infection strategy. In reality, the opposite is likely true: in an environment where opportunistic interaction determines the fate of a virus capsid, its structure and ability to successfully interact with an amenable host is of great importance. The study of their structures and mechanics goes hand in hand with progress on understanding their role in the global ecosystem. Indeed, the study of virus dynamics for the prevention and control of infections in both animals and plants (and the associated societal and economic devastation), has been popular ever since their discovery around a century ago. Understanding the viral mechanism and structure-function relationship is often a key focus for developing prevention and mitigation strategies. The techniques used in these studies are constantly evolving and improving, providing a greater insight into the hidden world of these tiny entities. Crucially, whilst applied virus research usually drives technological developments, marine virologists have been relatively quick to apply these techniques to their own model systems. Yet, in an increasingly competitive research environment, structural studies on marine viruses and their hosts could mistakenly be regarded as a low priority activity, especially in a still young field where easily accessible genomic studies continue to provide far reaching implications on broader virus function. Here we provide a brief introduction to the history of viral structural studies, the techniques and instrumentation involved, the mechanism by which data and theory pushes forward the knowledge and understanding. We also include a brief discussion on how the current work in some of these techniques might advance and develop in the near future, and how this can influence and may relate to future algal virus study.

### Early Virus Structural Studies

Virus structure knowledge took a leap forward in the year 1956. Crick and Watson suggested the hypothesis that ‘rod’ and ‘spherical’ viruses could be built from regularly packed identical sub-units. The basis for this idea was the high degree of order seen in X-ray photographs of crystallized plant viruses [5]. In this same issue, Caspar explained how for the Bushy Stunt Virus, the number of subunits by X-ray is a multiple of 12, most likely a multiple of 60, with chemical data suggesting up to 300 [6]. Work published in the same year used electron microscopy (EM) with shadowing, and the ordered subunit packing hypothesis to suggest icosahedral structure for ‘spherical’ viruses [7]. These three examples neatly display the underlying methods of viral structure investigation: Orthogonal approaches to instrumentation and methodology, and theoretical models. Throughout the years, although the resolution to which virus structure is deduced is constantly increasing, the research still relies upon these core principles.

Post-1956, research included the probable icosahedral structure of Tipula iridescent virus using EM with double shadowing [8], direct subunit visualization, organization and symmetry of Sericesthis iridescent virus [9], and ϕ6 lipid envelope discovery [10]. We will briefly skip past these and the initial discovery of virus like particles (VLPs) associated with eukaryotic algal species [11] to the next crucial juncture in algal virology with the introduction of culturable eukaryotic viruses [12]. This development, driven by the chlorella-chlorovirus system, meant obtaining the quantity of virus required for further study was no longer an impediment in these new model systems. Algal viruses are diverse in type and all require study, however laboratory propagation in many cases still presents a challenge. This ability to culture, however, subsequently paved the way for more detailed research into both algal viral structure and function.

## 2. Electron Microscopy

The main technique for ultrastructural studies in this early era was electron microscopy (EM) having been invented in 1935 [13]. This description can be split into transmission electron microscopy (TEM, developed first) and scanning electron microscopy (SEM). TEM provides greater resolution than light microscopy and SEM offers surface imaging of solid samples, allowing for visualization of extremely small viral particles (see [14,15] for a comprehensive review of the early development of EM and SEM, respectively). Insights into structural features of infection are not only discovered by microscopy (e.g., [16]). Molecular and other laboratory techniques in combination with the imaging analytical disciplines increase understanding of virus properties. For example, chemical composition can lead to information on surface protein modification and number, potential internal or external lipid membranes and their criticality in infection dynamics [17]. Sequence/proteomic analysis can predict the presence of structures such as channel proteins [18]. For algal viruses, the work on Paramecium bursaria chlorella virus-1 (PBCV-1) from the 1980′s onwards was at the forefront of intensive characterization through a variety of techniques as they became readily available for applied use.

### 2.1. Cryoelectron Microscopy

**Figure 1:**
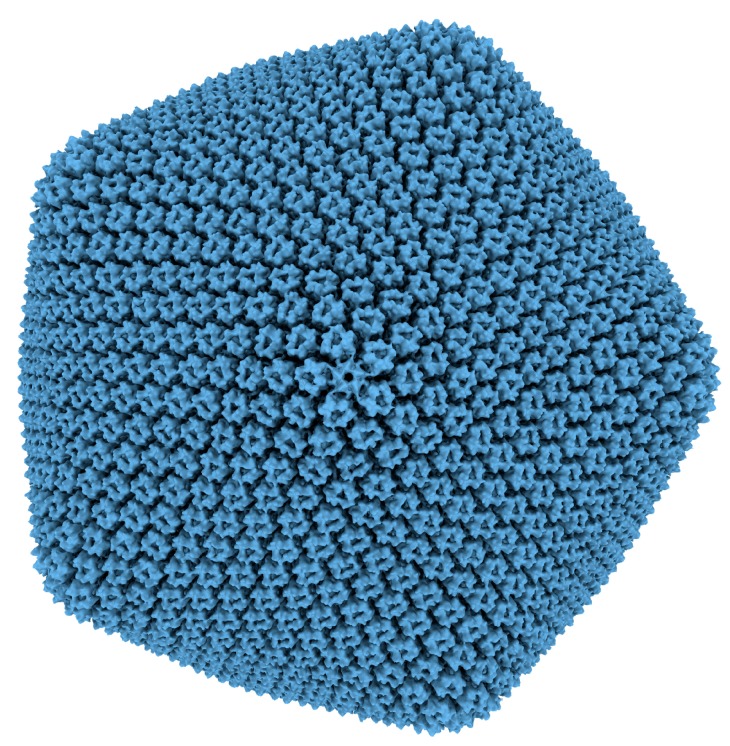
Cryoelectron microscopy map of Paramecium bursaria chlorella virus-1, used with permission and adapted from Zhang et al. [23].

EM received a huge upgrade for viral studies in 1984 with the publication of cryoelectron microscopy (cryo-EM) methods for virus samples [19] (see [20] for an interesting review of cryo-EM origins and beyond). For PBCV-1, 1996 was the year for cryo-EM analysis and 3D image reconstruction [21], with the technique providing impressive detail (26 Å resolution limit) into capsid construction and structure. From this data it was possible to ascertain the triangulation value (T) for the virus (T = 169 for PBCV-1). The T number of icosahedral viruses was first investigated in 1962 by Caspar and Klug [22] as a way to describe the 20T triangular faces of an icosahedron where T = Pf^2^ (P = h^2^ + hk + k^2^, for all pairs of integers h and k having no common factor; f is any integer). Using only basic electron micrograph and X-ray diffraction data the pair were able to generate a theory on virus structure using geometric rules, later proven correct by direct visualization. EM and cryo-EM data has been iteratively improving since its inception and use in icosahedral viral analysis, increasing resolution for PBCV-1 (Figure 1) three-fold to 8.5 Å [23] whilst using the T value theory as a way to describe structure.

### 2.2. Electron Microscopy Viral ‘Dynamics’

EM techniques are not only useful for intact virus structural study but can also be used in investigation of viral dynamics and the analysis of individual components. For example, when using TEM and its various derivatives such as scanning transmission electron microscopy tomography, immuno-electron microscopy and whole-cell focused ion beam-scanning electron microscopy [24,25]. These techniques give the ability to take a snapshot of an algal cell during infection; viruses will be in various stages of replication and assembly [26]. Analysis of this data can highlight the localization of viruses on the cell surfaces [27] and early infection events [28], location of assembly sites [29] and intracellular virus-host interactions [30]. This has peaked recently as examplified with single particle cryoelectron tomography (Cryo-ET) being used to investigate cyanophage-host interactions showing phage tail angles and membrane penetration [31]. Cryo-ET has also been used in combination with other techniques to analyze the herpes virus infection cycle [32]. Individual component analysis often combines EM data, with some form of theoretical modelling, sequencing and alignment to provide information (in the case of PCBV-1), on structure of the major capsid protein (MCP) and therefore capsomer subunits [33,34], functional surface channel proteins [18,35,36] and lipid membranes [37]. These techniques, although extremely powerful, currently cannot do justice to the dynamic nature of infection as they rely on individual static pictures in a constantly changing system. Observing this dynamism in real time is extremely difficult. Nevertheless, EM studies (SEM and TEM) continue to dominate the research landscape and are often the first tools applied to the structural study of previously uncharacterized viruses, often followed by the use of cryo-EM and other EM techniques.

## 3. Atomic Force Microscopy

**Figure 2:**
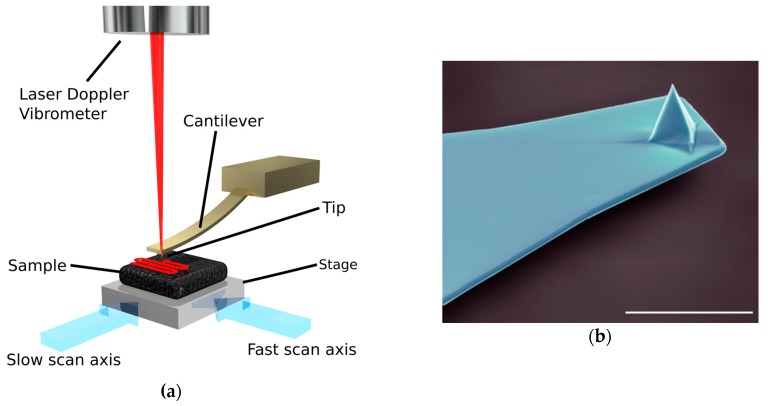
(**a**) Schematic diagram of a high speed-atomic force microscope created by O. Payton. Adapted from [42]; (**b**) Colourised electron microscopy image of an atomic force microscopy cantilever (scale bar 10 µm), the imaging tip is visible at the apex of the sharp, pyramidal structure at the free end of the cantilever. Image by Steve Gschmeissner.

Another form of microscopy and virus structure analysis tool is atomic force microscopy (AFM). Invented in 1986 by IBM scientists [38], the AFM relies on the measurement of deflection in a small cantilever as it passes over and physically interacts with a surface (Figure 2). This interaction can occur in a few different ways. Contact mode being the original imaging mode has the cantilever probe in continuous contact with the surface during scanning. Intermittent contact mode or tapping mode oscillates the cantilever at its fundamental resonance frequency with the probe interacting with the surface at the end of each oscillation. The cantilever can be monitored by, for example, optical beam deflection, optical interferometry or laser doppler vibrometer. Compared with the above-mentioned EM methods, and in the context of this review, AFM promises the ability to analyse biological samples without fixative steps, staining or labelling, in environments that are physiologically relevant (see [39] for early biological applications of the AFM). The average resolution for AFM ranges from sub nanometer to 50 nm depending on sample type, preparation and instrumentation. The primary negative of AFM is the slow speed of data acquisition, which is itself a result of the serial nature of data collection as the cantilever moves from pixel to pixel. With small image areas (typically less than 10 × 10 µm) and long collection times (typically between 5 and 10 min for a 512 × 512 pixel image collected at a scan rate of 1 or 2 lines per second) it is challenging to collect enough data when compared to EM studies. It was not long after its initial design that AFM studies were performed on viruses. By 1992, a group at IBM had published an experiment observing the biological, in situ process of pox virus infection of live cells [40]. This was achieved by holding the target cell in place with a patch-clamp pipette, while the cantilever deflection was measured using either a tunnel probe (i.e., from a scanning tunneling microscope) or the deflection of a reflected laser beam falling on a quadrant photodiode. The images provided revealed the cell membrane structure as well as time-course exocytotic processes. Notably, at the time of this study the atomic force microscope had only been available for roughly 10 years but had already exhibited the time resolution and imaging environments necessary to observe a dynamic event, albeit with slow image acquisition. A similar study was again performed in 1997 with increased speeds of one frame per second [41]. An impressive feat for the time and a hint of the future to come.

Although a cryo-atomic force microscope was developed in 1996 [43], this technique requires specialist equipment and doesn’t offer obvious advantage over EM and cryo-EM for virus analysis and has been largely ignored in studies. Interestingly, a review of marine virus study in 1997 [44] fails to mention the development of AFM and the potential for viral structural analysis compared to other microscopy methods. Perhaps suggesting that AFM at that time was a niche field, unknown and unavailable to many researchers. In 1999 a structural biology group from the University of California with experience in X-ray diffraction analysis of crystals reported one of the first examples using AFM as a tool for viral structural analysis [45]. This study used the crystallization of Turnip Yellow Mosaic Virus (TYMV, A relatively simple T = 3 tymovirus) and tapping mode AFM [46,47] as a platform to directly visualise the capsomere structures of virions immobilized within crystals. Presumably the group was successful due to the increased physical stability offered by immobilization, and reduction in cantilever-tip forces on the sample through tapping mode AFM. Although impressive, and a demonstration of the power in directly mapping a surface, this technique did not offer the advantages expected from AFM, namely viral investigation in, or at near physiological conditions due to limitations in viral preparation methods and knowledge at that time. The year of 2001 took the technology, in one aspect, closer to this goal, with the imaging of free virus directly adsorbed to mica substrate by AFM analysis in liquid [48]. Icosahedral viruses such as satellite tobacco mosaic virus, brome mosaic virus and cauliflower mosaic virus yielded capsomeric structure without the use of a crystalline lattice. Enveloped viruses tipula iridescent virus and herpesvirus did not immediately display capsid structure, but with a detergent treatment to remove their outer lipid membrane these were also imaged successfully. Rod shaped viruses such as tobacco mosaic virus were also imaged in a liquid environment. Being able to obtain quality data without crystallization of the virus sample is a huge advantage for AFM as an analysis tool. This doesn’t, however, mean that AFM requires no sample preparation, with immobilization of the sample being key to successful imaging [49].

### 3.1. PBCV-1 Atomic Force Microscopy

**Figure 3:**
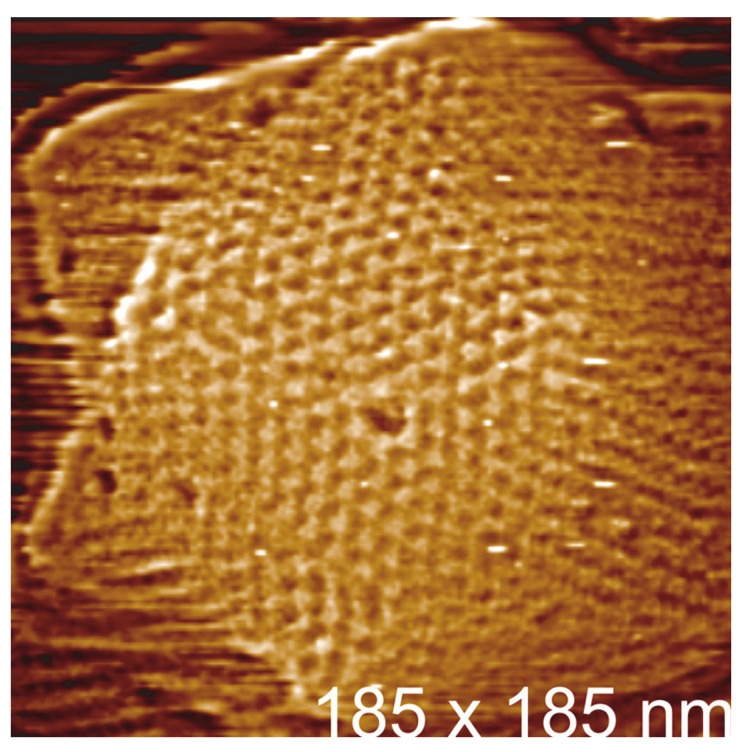
Selected AFM image of PBCV-1, used with permission from Kuznetsov et al. [51].

For the next twelve years the California group published extensively their research into AFM study of viruses (reviewed in [50]), with the introduction of algal viruses in 2004 [51] as seen in Figure 3. It is mentioned in this study that due to the large size of PBCV-1 and its softness, for AFM imaging a glutaraldehyde fixation step and a poly-L-lysine treatment of the mica surface was required, with the structural data obtained intended to complement the electron microscopy and X-ray diffraction analyses that preceded it. For algal virus structural studies this was a demonstration of technique applicability, and further highlights PBCV-1 as the premier test subject in this realm, but in some respects could be interpreted as not offering advantage over the established microscopy methods. This is not entirely true (mentioned by the authors) as AFM is not limited by both symmetry averaging (cryo-EM) or physical averaging (crystallization) and can also give detailed biological defect and anomaly examples, including an insight into internal structural features when imaging damaged and imperfect particles.

### 3.2. Atomic Force Microscopy Viral ‘Dynamics’

It is of note that a 2002 study by the California structural group performed a similar viral infection experiment to the IBM scientists from 10 years previous [52], however, in this instance the group relied on heavy dehydration, fixation and post-fixation to provide extremely high quality imaging of murine leukemia virus (MuLV) emerging from NIH 3T3 cells. Similarly, the same technique was applied to Human Immunodeficiency Virus (HIV) and lymphocytes, in the process providing the most detailed cellular AFM data at that time [53]. This method has similar restrictions to this style of study when utilizing EM and cryo-EM, in that only an individual moment of infection is visualized. In 2007 a similar publication examined a MuLV mutant with the same methodology, with no image acquisition speed increase (approximately 4 min per frame) [54]. Fixation at differing time points and having virus naturally at various stages of infection can act as proxy to dynamism. but even though the resolution of the techniques was extremely impressive, the ‘real-time’ measurement of virus infection initially reported in 1992 was still elusive. In the year of 2008 a comparable study was performed showing an increase in resolution with less reliance on fixation and dehydration and an image acquisition interval of 6 min for Moloney murine leukemia virus (MLV) budding events [55], but maybe not displaying the expected potential improvements from over a decade of progress.

### 3.3. Improving AFM Speeds

As with EM before the invention of cryo-EM, the technical challenges of improving the instrumentation and methodology was restricting the key theoretical output of what AFM should be able to offer. A few groups were working in the late 90s/early 2000s on one facet of this limitation, the speed of imaging. It was at this time high speed-atomic force microscopy (HS-AFM) emerged in its early state after being initially described in 1991 [56]. Development of key speed increases were demonstrated via observations of biopolymers, biomolecules and soft crystalline or molten crystalline polymers. This led to increases in single frame acquisition time from four to six minutes to 1.7 s [57] and within six years, framerates of 12.5 s^−1^ [58] and 70 s^−1^ [59], to essentially provide video data via HS-AFM. It should be noted that contact mode HS-AFM was developed for materials science as opposed to the more classically biologically relevant tapping mode [60], but, due to imaging orders of magnitude faster, offers reduced probability of sample damage compared with conventional contact mode AFM [61] and frame rates as high as 1300 frames s^−1^ [62]. With this significant increase in image acquisition speed, the stable foundations for dynamic, viral process study were laid.

Perhaps one of the most prolific groups in AFM is the Bio-AFM frontier research center at Kanazawa University. Becoming particularly well-known with the 2010 *Nature* publication of myosin V translocation along actin filaments by HS-AFM imaging [63] and recently successfully visualising the dynamics of a CRISPR-Cas9 system in real time [64]. However, these studies are likely the tip of the iceberg regarding their HS-AFM progress. Described in 2001, 2002 and 2003 using myosin V as the test sample, the group explained their new tapping mode HS-AFM. This had an image acquisition time of 80 ms and data showing potential for collecting video data of molecular processes in buffer in real time [58]. Progress has continued for the past 15 years focusing on the applicability of HS-AFM to molecular mechanisms which is reviewed extensively in 2008 [65]. The limitations to imaging speed and examples of biomolecular process study were explained in 2012 [66], 2013 [67], 2014 [68] and 2017 [69,70], respectively. In the most recent review, the future of HS-AFM is discussed speculating on faster imaging rates, hybrid HS-AFM/optical microscopy or optical tweezers systems, nanoendoscopy and noncontact imaging. Although viruses, and specifically algal viruses, are not mentioned the improvement in the technology associated with this ‘soft-touch’ (low interaction forces) methodology HS-AFM could certainly be applied in monitoring interactions between viruses and cell surfaces. For example, using methods such as generating artificial membranes in order to visualize porating proteins [71,72] could be used to investigate the molecular bio-machines that might be involved in cell adhesion and entry or imaging the reconstructed or in situ ionic channels predicted in algal viruses [73].

An alternate strategy for improving the speed of HS-AFM to the optimized tapping mode developed by researchers at Kanazawa University utilizes contact mode HS-AFM with low spring constant AFM cantilevers and a passive mechanical feedback loop [59]. This format of HS-AFM removes the bottleneck associated with developing faster feedback electronics and mechanics and has the highest potential imaging speeds [42]. The loss of force regulation does however make the technique unsuitable for samples requiring extremely low interaction forces. Contact mode HS-AFM also offers the ability to perform large area scans at high resolution. The biological variance expected in algal virus systems could be accommodated with better sample statistics. Rare events are more likely to be discovered and easier to monitor. For instance, contact mode HS-AFM has recently been used to quantify amplicon expression and contamination [74,75], and CRISPR-Cas9 sequence targeting DNA mapping [76]. This suggests potential for HS-AFM (when combined with previously mentioned subsurface AFM techniques and the ability to analyze large sample areas) in analysis of internal viral or cell features before, during and after algal infection.

The obvious step for HS-AFM in algal virology is to move towards not only imaging of the virus for structural information, but the interactions involved in infection to understand the dynamics between the virions and the cells they infect. One part of this interaction that has only been briefly mentioned thus far and must be acknowledged is the AFM imaging of the cell itself. Whole and intact cells offer many difficult challenges as samples for AFM. AFM relies on tip interaction with the surface for data acquisition. Cells are essentially large, biological, liquid filled, membrane surrounded ‘balloons’ with a tendency to be damaged by prolonged interactions with the AFM cantilevers. Even with this limitation, the obvious advantage of AFM of cellular structures was soon realised and proved possible by the 1992 contact mode, in situ, virus infection study [40]. The investigation of granule motion, membrane spreading in human platelets [77]; and other studies also utilized AFM for cell imaging (for the early review into cell imaging by AFM see [78]).

**Figure 4:**
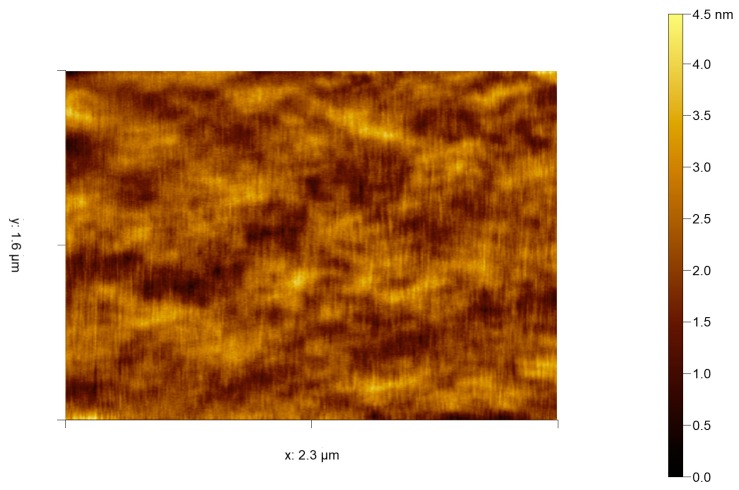
Selected contact mode HS-AFM frame of *Emiliania huxleyi* cell surface captured at 0.5 s per frame by the authors. Variations in height are likely due to membrane ruffles, localised variation in membrane lipid composition/size and their associated membrane proteins.

**Figure 5:**
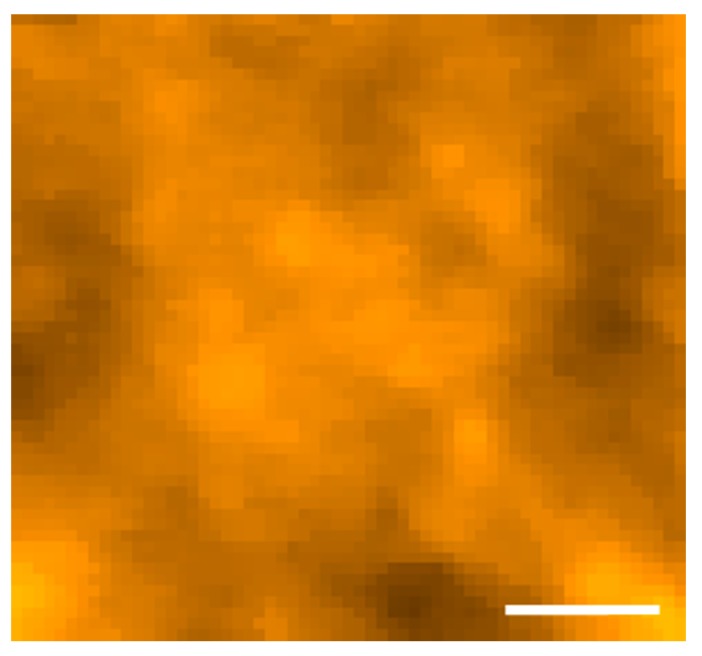
Selected tapping mode HS-AFM frame of COS-7 cell surface captured at 6 s per frame, used with permission from Shibata et al. [84]. (max height 45 nm, scale bar 500 nm) This is a single image from a series that shows membrane pit closure over 78 s.

The development of tapping mode as an improvement to AFM for biological studies has already been mentioned but applies even more so to the softness of cells. Physical fixation of samples as big as cells is also an issue for AFM, with the instrumentation having difficulty in dealing with the large height differentials seen in many cells. One can only imagine the practical difficulty in the patch clamp method used by the pioneering IBM group, fortunately a more user-friendly method was described in 1995 [79]. Here, *Saccharomyces cervicae* yeast cell suspension was pushed through a 5-µm (roughly cell diameter) pore size Millipore filter using a modified syringe system. This caused the cells to become trapped in the filter with only the top-most dome of the cell protruding above the filter surface or having a far lower height differential between cell and filter surface available for imaging. This idea was again revisited in 2015, albeit with a custom and updated construction method (photolithography) of the cell fixing array [80]. For algal studies, to date, the main interest has been in the in vivo study of diatoms, mainly due to interest in biomimetics of the diatom silicified frustule [81,82] and also secreted adhesives such as by the green alga *Enteromorpha* [83]. Although several studies have been made into visualizing infected cells (often mammalian) with various viruses, to date there have been none involving algal viruses. Advances into algal cells and virus have very recently been made with an example presented below in Figure 4. Here we present select contact mode HS-AFM data of the *Emiliania huxleyi* algal cell membrane surface collected at 2 frames per second. This is then compared to the current very best in tapping mode HS-AFM data of a mammalian COS-7 cell membrane (Figure 5) [84] showing comparable resolution and detail of surface topography. Both figures show images that are state of the art, collected on live cell membranes. Whilst neither captures any obvious features this is an issue with showing single images from ‘movie’ data. The key benefit that HS-AFM techniques bring is that their resolution is the same as regular speed AFM and the quick succession of images reveals minute fluctuations and events on the cell surface. Single images simply don’t do justice to the resolution because without the other frames it is too difficult to identify signal from noise and fluctuations from actual structure. Since the development and invention of AFM, the capabilities of the technique have consistently improved and are now reaching a point where the original premise and potential of AFM can be realized. It is the authors’ opinion the current lack of studies is both due to the difficulty of live cell imaging by AFM, the lack of instrumentation availability at marine focused institutions and the interest in algal viral dynamics.

To measure these algal host-virus interactions, there are currently two main approaches that could be pursued. One is taking advantage of the speed of HS-AFM to truly visualize and gather data on dynamic infection processes. To achieve this, taking advantage in improvements in live cell imaging techniques and using a HS-AFM with the resolution and frame rate necessary to observe fast biological processes is key. The second avenue is an aspect of AFM not thus far mentioned. Atomic force microscopes can be operated in a point-measurement mode that enables them to collect force-distance curves at different locations on the nanoscale surface. These curves can then be processed to obtain data about the material properties of the sample surface. With these methods it may be feasible to quantify lipid domains/sphingolipid raft size structure and characteristics with conceivable inference of sphingolipid production rates in examples such as *Emiliania huxleyi* in natural and modified forms. Also, discovery of specific membrane areas involved in algal viral adhesion, and fusion entry and exit should be possible.

### 3.4. Force-Distance Curve-Based Atomic Force Microscopy

Having the sensitivity to measure piconewton interaction forces enables AFM to study the interaction forces between single biomolecules [85] and to probe microbial cell surface properties. This includes charge, hydrophobicity, elasticity and receptor ligand interactions [86]. This technique has been recently reviewed in relation to microbial cells [87] with the author questioning the utility of HS-AFM for live cell imaging, mentioning the limitations of whole cell imaging. We believe the technology will, in time, overcome these methodology and instrumentation issues. This aspect of AFM (sometimes called force spectroscopy) deserves far more discussion, but in the context of this review the most important piece of work to date involves using AFM cantilever tips functionalized with a PEG crosslinker and bound to an engineered rabies virus in combination with confocal microscopy. With this ‘activated’ tip, an interaction force map was generated of the live cell surface by measurement of the interaction forces and therefore likely areas of adhesion between the virus and cell in various locations [88,89]. This exhibits the possibility for using this technique in other virus-host systems and could, with some work, be applied to algal virology (likely involving a combination of techniques from a PBCV-1 dynamic attachment study [90] and the afore mentioned AFM force spectroscopy) as a useful form of AFM in examining viral binding and entry dynamics.

There are other imaging modes available for AFM [91] that have potential in algal virus study and must be mentioned. For example, multifrequency AFM that can provide more information by measuring several frequencies of cantilever motion such as simultaneous measurement of topography and the viscosity map of virions [92], cells [93] and VLPs [94]. It is also possible to use the AFM as a physical micromanipulator in force nanoindentation studies to map the mechanical properties of viruses in uncoating and capsid interaction studies [95,96] and there exists a modification of force spectroscopy using hybrid binding domain functionalised AFM cantilevers to investigate and quantify the in situ hybridization of miRNA and other nucleic acids in single cells [97].

### 3.5. Subsurface Atomic Force Microscopy

**Figure 6:**
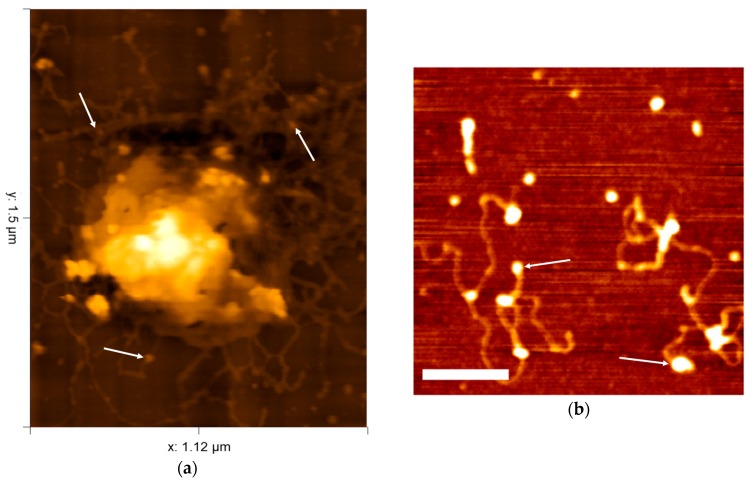
(**a**) Selected contact mode HS-AFM frame of disrupted Emiliania huxleyi Virus 86 spilling its genomic content captured at 0.5 s per frame by the authors (max height 37.1 nm); (**b**) Selected AFM image of PBCV-1 DNA pre proteinase K treatment, used with permission and adapted from Wulfmeyer et al. [106] (scale bar 100 nm, max height 2.4 nm). Arrows show ([a]-putative) DNA associated proteins.

AFM, in its basic form is fundamentally a surface imaging technique similar to rudimentary SEM. SEM in truth does have an interaction volume, giving information about electronic and elemental characteristics. AFM can provide physical and mechanical characteristics of samples such as friction and stiffness. However, in the context of this section SEM and AFM are considered as surface imaging techniques. The idea that AFM in this respect provides insight into the internal features of various biological samples seems counter-intuitive considering the mechanism of data acquisition. Indeed, few studies have explored this idea. With current technology, techniques including Cryo-ET, confocal and fluorescence based microscopy arguably produce better results with less effort. However, some intriguing progress has been made into using AFM to examine the structures hidden below the surface of viruses. As eluded to above, in the case of virus structural study, the disruption of the capsid shell and in some cases external lipid membrane either by accident or on purpose, often exposes the features not immediately available by AFM or SEM as seen in Figure 6a. The initial, and arguably most influential, work in this area was seen in 2002 using Herpes Simplex Virus-1 (HSV-1). Using various methods involving differing levels of vigor of detergent treatments causing removal of external lipid envelopes to expose capsid structure, tegument proteins, the structure of the lipid envelope itself and even the DNA and its associated proteins were observed spilling out from the core of the virion [98]. They predicted that AFM resolution would improve in the future to match the resolution of EM. The same group applied similar detergent based treatments to viruses such as Intracellular Mature Vaccina Virus (IMV) in 2003 in conjunction with enzyme proteinase K treatment to directly examine the internal DNA genome structure and its surrounding 30–40 nm diameter tubules [99], and the RNA of HIV [100] and TYMV [101]. In an extension of this idea, AFM was used to examine phenol/chloroform extracted RNA from several icosahedral viruses to display consistent structural dynamics of the RNA during preparation and imaging [102]; highlighting potential for structural analysis of nucleic acids in both its ‘natural’ and extracted forms, and in parallel with other biophysical analysis [103]. Another improvement in methodology was seen in 2006, utilizing ribonuclease A to discriminate between single- and double-stranded DNA and RNA [104]. Using specific enzymes and antibodies and other obviously noticeable sized proteins to highlight certain structural features is an important method in AFM data confidence, considering the usual lack of markers. Perhaps the most extensive use of these ‘internal features’ AFM techniques was in the investigation into Mimivirus viral factories. Mimivirus replicates in the cytoplasm of amoeba, offering unparalleled insight into its replication cycle through direct visualization of these locations [105]. The field of algal virology first invoked these methods in 2012 in combination with a fluorescence-based approach (4′,6-diamidino-2-phenylindole [DAPI] DNA stain) [106]. PBCV-1 releases its DNA for fluorescence imaging into the media during high multiplicity of infection of *C. variabilis* cells and AFM of osmotic shock treated virus was used to examine single virions and their emerging DNA with associated proteins (Figure 6b). Further treatment of the exposed DNA with proteinase K removed the proteins and it was possible to calibrate AFM images by analysis of BSA and a purified, putative DNA-binding protein coded by PBCV-1 to measure the size of DNA associated proteins. Using the structural data and other molecular techniques, hypotheses were generated regarding DNA packaging within the virion and behavior during infection. It is worth mentioning that DNA has also been imaged by AFM to very high resolution revealing different structural conformations of the DNA double helix [107]. Current AFM studies in this area are limited, being performed under nonphysiological or destructive conditions. Other techniques currently provide arguably biologically significant data regarding infection monitoring. Although the use of AFM for internal imaging is an initially, unexpected output, and again currently lacks the dynamic nature of infection, it shows potential for future work into algal viral dynamics when potentially combined with different methodology and improved instrumentation.

## 4. Conclusions and Perspective

The focus of algal virus study has followed the same paths as generally pioneering studies into medically relevant, human pathogenic viruses. This being the initial interest into the unseen structure of these nanoscale biological particles, how their structure relates to and follows geometric rules, the structure of the individual building blocks and evenually insight into infection dynamics and strategies. The principles outlined early in this review of theory and orthogonal instumentation and methodology have been key in every stage of the improving understanding. Theory suggests hypotheses to later be proven by improved instrumentation, and the improved instumentation providing data from which more detailed theory can be hypothesised. Key to this process, and outlined in part in this review, is the constant iteratively refining methodology for analysing these host-viral systems on such a small scale. We have covered the basic history of some of the methods available for use in experimental studies at this time, with a particular focus on different forms of microscopy and especially atomic force microscopy. Atomic force microscopy, we believe, has potentially been overlooked as purely a structural tool, but has showed recent promise as a method for dynamic observations. We have confidence that with reducing costs and increased availablity and when applied in parallel/correlatively with other virology approaches; these techniques can be effectively functional in the study of algal viruses to determine replication mechanisms and to collect evidence for existing theory. Hopefully, as is usually the case, once methods are established they become far easier to perform and more widely available, leading to a far wider range of algal viruses being studied in greater detail when compared with the limited test systems being used in recent histroy and current work.

What is the limit of resolution when using these techniques? For EM, we are currently at near atomic resolution [108] and for AFM it is possible to resolve molecules [109,110] and collect data at the atomic scale [111,112]. We believe AFM to be of extreme importance as a tool for studying the host, virus and the infection process in a dynamic way that is not applicable to other approaches. In the future, who knows how far we can push our understaning of these important and globally relevent viral systems? One thing is for certain, the study of algal viruses has a great future.
